# Comparison of Growth Patterns of COVID-19 Cases through the ARIMA and Gompertz Models. Case Studies: Austria, Switzerland, and Israel

**DOI:** 10.5041/RMMJ.10413

**Published:** 2020-07-31

**Authors:** Francisco Javier Diaz Perez, David Chinarro, Rosa Pino Otin, Ricardo Díaz Martín, Moises Diaz, Adib Guardiola Mouhaffel

**Affiliations:** 1 Faculty of Health Sciences, University San Jorge, Zaragoza, Spain; 2 Faculty of Engineering, University Distancia Madrid (UDIMA), Madrid, Spain; 3 Department of Computer Science, University Atlántico Medio, Las Palmas, Spain

**Keywords:** ARIMA, coronavirus, COVID-19, Gompertz, growth model

## Abstract

On May 19, 2020, data confirmed that coronavirus 2019 disease (COVID-19) had spread worldwide, with more than 4.7 million infected people and more than 316,000 deaths. In this article, we carry out a comparison of the methods to calculate and forecast the growth of the pandemic using two statistical models: the autoregressive integrated moving average (ARIMA) and the Gompertz function growth model. The countries that have been chosen to verify the usefulness of these models are Austria, Switzerland, and Israel, which have a similar number of habitants. The investigation to check the accuracy of the models was carried out using data on confirmed, non-asymptomatic cases and confirmed deaths from the period February 21–May 19, 2020. We use the root mean squared error (RMSE), the mean absolute percentage error (MAPE), and the regression coefficient index *R^2^* to check the accuracy of the models. The experimental results provide promising adjustment errors for both models (*R^2^*>0.99), with the ARIMA model being the best for infections and the Gompertz best for mortality. It has also been verified that countries are affected differently, which may be due to external factors that are difficult to measure quantitatively. These models provide a fast and effective system to check the growth of pandemics that can be useful for health systems and politicians so that appropriate measures are taken and countries’ health care systems do not collapse.

## INTRODUCTION

In late 2019, several cases of pneumonia with unknown characteristics and syndromes were detected in China,^1^ the causative agent being a new coronavirus, now called severe acute respiratory syndrome coronavirus-2 (SARS-CoV-2),^2^ which generates a disease called COVID-19 (COrona VIrus Disease of 2019). This new coronavirus has been found to have 88%–96% sequence similarity to bat coronaviruses in its genome, according to other pathogenic coronaviruses such as SARS-CoV and MERS-CoV.^3^,^4^ This new SARS-CoV-2 coronavirus is a pathogen that has shown a high potential for contagion,^5^,^6^ with person-to-person transmission. The primary way the virus disseminates^7^ seems to be close contact between individuals, with transmission by respiratory droplets spread by coughing or sneezing.^8^ Moreover, transmission occurs primarily through aerosol concentrations in confined areas.^9^ Current data suggest that the virus has an incubation period of 3–7 days, with an average period of 5.2 days.^10^ Other authors indicate periods between 2 and 14 days,^11^ and there are even reports of cases with an incubation period of up to 24 days.^12^ The rapid spread of the disease is explained by the ability of the virus to survive several days outside a host,^13^ highlighting an active persistence on metals and glass of up to 9 days.^14^ Given the severity of the outbreak and its rapid spread, the World Health Organization (WHO) declared the new disease as a public health emergency of international concern on January 30, 2020.^15^ On March 11, the spread had reached 118,000 cases in 114 countries, with 4,291 people dead, and the WHO Director-General declared COVID-19 a global pandemic.^16^ On May 19, the situation was 4,731,458 confirmed infected, 316,169 dead, and 180 countries affected by the epidemic.^17^ Subsequently, the coronavirus has spread worldwide at an alarming rate, causing more infections and deaths than previous SARS or MERS coronaviruses.^18^

The high speed of transmission of the virus has generated multiple preventive and control strategies by different countries. Indeed, governments have had to apply new measures urgently to their countries, such as isolation, screening tests, or prophylactic measures. Although governments managed to flatten the epidemic curve a little, they have not been able to prevent the spread of the virus by other countries of the world. One possibility for dealing with this is through mathematical models. There is an urgent need to develop mathematical models that allow predicting the behavior of the pandemic in the different affected areas. It would lead to taking the most appropriate prevention and control measures for the outbreak in each circumstance and with enough time in advance. Modern mathematical epidemiology can be considered to have started with the studies of Kermack and McKendrick with the classical model of susceptible–infected–recovered (SIR).^19^ Subsequent investigations for outbreaks of SARS^20^ and cholera^21^ apply more complex models with multiple variants, such as the susceptible–infected–recovered–susceptible pattern (SIRS),^22^ where patients who have recovered can be reinfected due to not developing immunity; or the susceptible–exposed–infectious–recovered model (SEIR),^23^ where the exposed population is evaluated, and resistance is improved. Thus, a logistic model has been applied to describe the growth and development of diseases. This model can be seen in the case of bacterial growth^24^ and in the spread of infectious disease.^25^ Also, the Gompertz model has been used to model bacterial outbreaks.^26^ The automatic regressive integrated moving average (ARIMA) model has been successfully applied in the field of health care to estimate the incidence and prevalence of mortality from malaria,^27^ hepatitis,^28^ and other infectious diseases.^29^,^30^ Currently, the studies that have been carried out on the growth of COVID-19 have focused on specific areas, mainly in China. Examples include the SEIR model of Wu et al.^31^ that predicted the national and international spread of the pandemic, and the study of Yang and Wang,^32^ which introduces a lockdown variable. Logistic models have also been used successfully to predict infections in China.^33^ They have also been used to analyze the temporal dynamics of the disease in China, Italy, and France^34^ and the growth in the number of those infected in Iran.^35^

The objective of this study was to obtain a model capable of predicting behavior (number of infections and mortality). This would assist health managers and politicians in predicting situations for better decision-making in the control and prevention of this pandemic. Our proposal is based on mathematical models of the spread of infectious diseases since they are essential to understand the evolution of epidemics over time. We have taken data from countries with similar numbers of habitants in Europe (Austria and Switzerland) and Asia (Israel). Then, we apply a growth model based on the Gompertz function and use the ARIMA statistical model for validating the application in the growth forecast of this pandemic. We outline our paper as follows. First, we present our methodology where the procedure is explained. Next, we describe both the Gompertz and the ARIMA models, followed by the calculations and prognostic models for deaths and infections. We close the paper with the conclusions of the investigation.

## METHODOLOGY

The investigation of the growth prediction of the COVID-19 pandemic was carried out in three countries with a similar number of habitants, namely Austria, Switzerland, and Israel. To this aim, we compare the results obtained with Gompertz’s growth model and the Box–Jenkins ARIMA statistical model. All three countries took similar lockdown measures, border closures, restriction of movement, and closure of shops; the most restrictive were Israel and Austria, while Switzerland’s rules were the least restrictive for its citizens. All three countries are now beginning the easing of lockdown of its citizens and the opening of trade. Relaxed confinement measures were first made in Austria, starting April 14, then Israel on April 16, and, finally, Switzerland on April 27. The measures taken have been effective differently in each country, as can be seen in Table 1A.^36^–^39^

**Table 1:** Summary of Infected and Mortality Data by Country.

Data	Austria	Israel	Switzerland
**A:** Summary of data by country, updated on May 19, 2020			
Population	8,800,000	8,600,000	8,600,000
Population density (people/km^2^)	105	390	208
Life expectancy	82	83	84
% Population aged >65	20%	12%	19%
Case fatality rate (CFR)	3.9%	1.7%	6.2%
**B:** Summary of current (May 19, 2020) and predicted values of COVID-19 infections			
Current infected (May 19)	16,321	16,659	30,618
Infection prediction	16,500	17,200	31,000
Infection prediction/10^6^ population	1,875	2,000	3,605
Forecast pandemic end date	June 2020	July 2020	July 2020
**C:** Summary of current (May 19, 2020) and predicted deaths values			
Current deaths (May 19)	632	278	1,891
Forecast deaths	650	300	2,000
Case fatality rate (CFR)	3.90%	1.70%	6.50%
Forecast death/10^6^ population	74	35	233
The end date of pandemic deaths	June 2020	June 2020	July 2020
**D:** Summary of the error and adjustment coefficients of the models obtained from those infected			
Gompertz method:			
Growth coefficient	0.097	0.087	0.085
RMSE	472	307	459
MAPE	10.05%	17.64%	12.82%
*R^2^*	0.9972	0.9993	0.9994
ARIMA method:			
ARIMA (p,d,q)	(0, 2, 4)	(0, 2, 1)	(1, 2, 0)
RMSE	130	141	182
MAPE	3.52%	4.92%	5.44%
*R^2^*	0.9998	0.9998	0.9997
**E:** Summary of the error and adjustment coefficients of the models obtained from mortality			
Gompertz method:			
Growth coefficient	0.083	0.074	0.078
RMSE	9	8	22
MAPE	5.29%	9.39%	7.26%
*R^2^*	0.9996	0.9976	0.9996
ARIMA method:			
ARIMA (p,d,q)	(2, 2, 0)	(0, 2, 1)	(1, 2, 2)
RMSE	5	3	14
MAPE	8.11%	6.29%	5.03%
*R^2^*	0.9998	0.9997	0.9998

These data evidence that the most efficient systems for isolation/lockdown, at the moment, were implemented by Austria and Israel, with an infection rate of 1,855 and 1,937 people per million inhabitants, respectively. In contrast, Switzerland reached an infection rate nearly twice that of Austria and Israel, with 2,560 cases per million inhabitants. Regarding deaths, it can be seen that the highest mortality rate was in Switzerland, where they had a death rate of more than 6.2% of those infected, and a per capita mortality of 220 deaths per million inhabitants. The country with the lowest mortality rate is Israel, with a 1.7% mortality rate and 32 deaths per million inhabitants.

**Figure 1:**
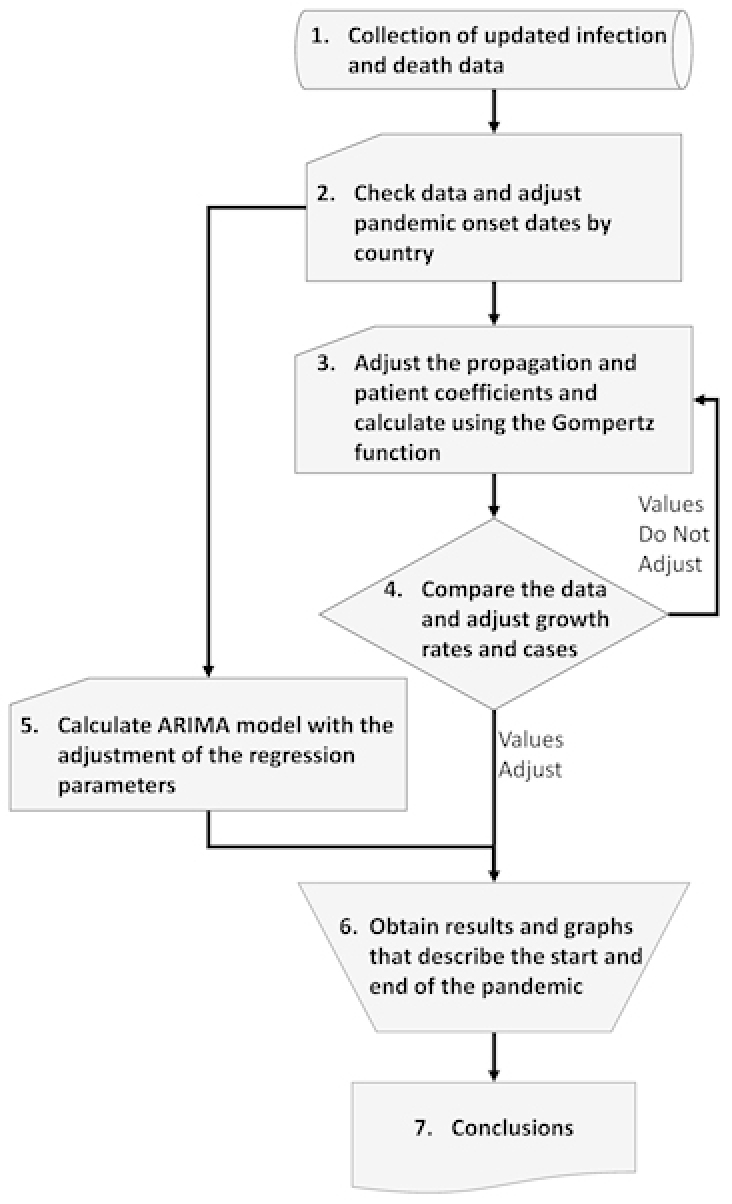
Overview of the Research Flowchart.

In this paper, we report on a study of the increase in numbers of infected and dead due to COVID-19 using the Gompertz growth model and the ARIMA statistical model. Specifically, to obtain the growth forecasts we employed the following procedure (summarized in Figure 1):

1. Collection of data on infected people detected by tests and deaths from each country of the investigation until May 19, 2020.
2. Verification of the data and adjustment of the initial pandemic and death rates per country to make the calculations.
3. Adjustment of the assumed values of the growth, spread rate, and the maximum number of predicted cases of infection and death by country; calculation of the number of infected and dead individuals in each country using the Gompertz growth function.
4. Comparison between the calculated and the real data, adjusting the growth coefficients and the forecast of the cases, to get the values that best fit the actual situation; verification of the validity of the data collected by calculating the *R^2^*, root mean squared error (RMSE), and mean absolute percentage error (MAPE) values for each estimate and country.
5. Performance of the statistical calculations of the ARIMA model with adjustment of the regression parameters; verification of the validity of the data obtained by calculating the *R^2^*, RMSE, and MAPE values for each estimate and country.
6. Construction of a sigmoid graph describing the growth in number of infected and dead, and the estimated end of the pandemic for each country; to this aim, we used the predicted values that best fit the reality of each country.
7. Concluding description of more precise methods to contain future pandemics.

### ARIMA Method

The ARIMA method attempts to forecast the values of a variable by using only past observations. To extract the observed patterns, the structural conditions that make up the series, such that it remains constant, should be satisfied. This is known after its creators as the Box–Jenkins model.^40^,^41^ It is widely used for the analysis of economic series, as well as in hydrology or medicine.^42^,^43^ However, the field in which the ARIMA methodology finds its central role for prediction purposes is with short-term prediction and in series with a seasonal component.^44^ The ARIMA model provides a general methodology for the analysis of a single variable in the series that shows a clear dependence between the present and past values.

The generic name ARIMA derives from its three components: autoregressive (AR), integrated (I), and moving averages (MA). The ARIMA model presents an explicit equation that allows us to describe an observation of the series as a linear function of previous data and errors due to chance. Moreover, it can include a cyclical or seasonal component that describes each of the elements that can be part of the model, as well as the notation generally used to describe them, which is used in this study. The general function^45^,^46^ represented by the ARIMA model *(p, d, q)* is defined as follows:

(Eq. 1) $$\emptyset (\beta )\;{\left(1-\beta \right)}^{d}\mathrm{Xt}=c+\theta (\beta )ɛt$$

where *Xt* is the variable to study, *c* is a constant, and *ɛt* is the error or residue term, which follows a normal distribution of zero mean and constant variance. The term *(1-β)^d^* is applied to the original series to make it stationary, and *d* corresponds to the order of part *I* of the ARIMA model. *Ø(β)* and *θ(β)* are polynomials of order *p* and *q* that depend on the delay operator *β*.

### Gompertz Model

The other mathematical model that we have used to compare the growth forecasts of the pandemic is the Gompertz model, which belongs to the family of sigmoid curve modeling.^47^ There are different types of Gompertz curves depending on the parameters that compose them, but they all have a double exponential as a common characteristic element. With this function defined for human mortality, Charles P. Winsor^48^ began to study the growth of biological phenomena. He proposed the Gompertz model, which was later used by many authors in growth studies of all kinds.^49^–^51^ There is a multitude of options when it comes to expressing the Gompertz curve model because this name was assigned to a wide variety of curves, which have in common being double exponential. We use the following model to assess the growth and development of COVID-19:

(Eq. 2) $$\begin{array}{l} f(t)=k^{\left\{\left(ln^{\left(\frac{X_{0}}{k}\right)}\right)\;(e^{-\alpha (t-t_{0})})\right\}},\\ t\geq t_{0},\;\;\;\alpha >0,\;\;\;k>X_{0}>0 \end{array}$$

Considering *k* the maximum predicted number of patients infected or dead in the development of the pandemic, *X_0_* is the number of initial patients, infected or dead, when the epidemic starts at time *t_0_*. We also consider *t* the prediction time, and *α* is the growth rate characteristic of the pandemic. For biological growth calculations, we restrict the values of *t* (*t≥t_0_≥0*) and the initial number of patients *X_0_=f(t_0_)>0*.

This sigmoid curve is limited in time, shows a monotonous increase, and presents an inflection point. The curve changes from concave to convex at this point, reaching approximately 37% of the growth. The inflection point depends on *X_0_* and can be defined for *k>X_0_:*

Inflection point dimension (IPD):

(Eq. 3) $$\mathrm{IPD}=\left(\frac{\ln\frac{k}{X_{0}}}{b}+t_{0},\frac{k}{e}\right)$$

and the approximate percentage of growth at that point (APG):

(Eq. 4) $$APG=37\times \left(1-\frac{X_{0}}{k-X_{0}}\right)\%$$

**Figure 2:**
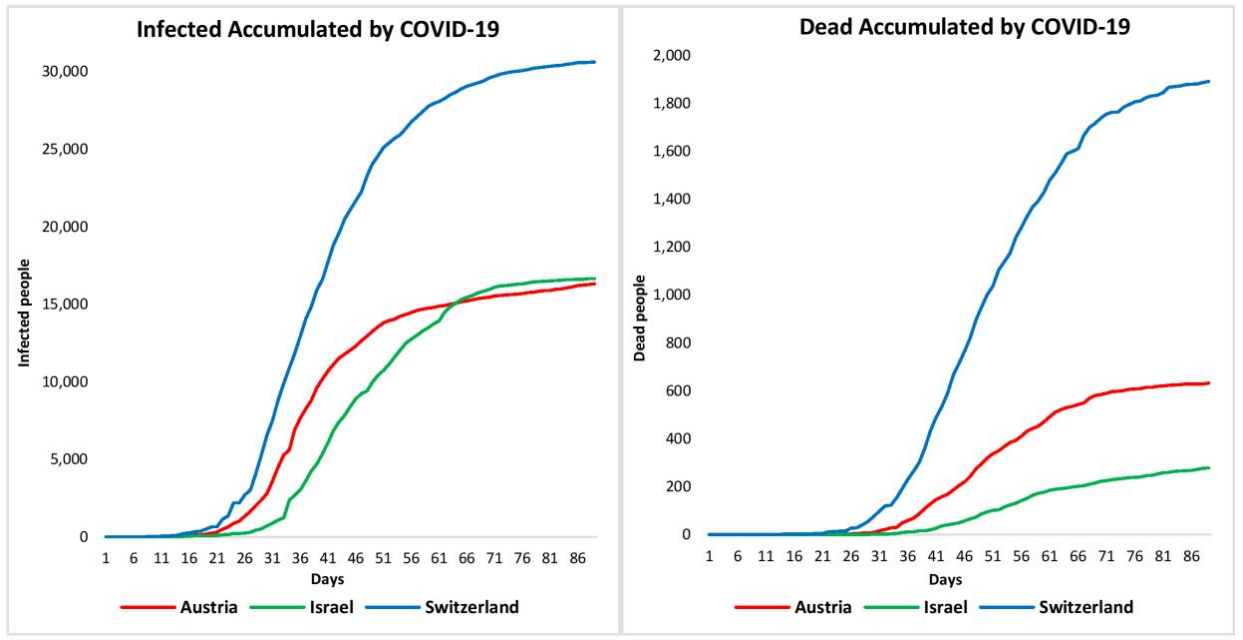
COVID-19 Data from Infected (left) and Dead (right).

Data on confirmed cases and deaths were obtained from the WHO,^37^ with daily reports submitted worldwide from the European Center for Disease Prevention and Control (ECDC).^39^ We consider the data of infected individuals from February 25, 2020, for all three countries. On the other hand, the day of the first death from COVID-19 was different for each country, namely March 5 for Switzerland, March 12 for Austria, and March 21 for Israel. The cumulative curves of deaths and infections for the study countries are illustrated in Figure 2.

We performed the forecast calculations for the Gompertz model and the ARIMA model with the data obtained of the numbers of infected and dead. Based on the mathematical modeling software IBM SPSS Statistics,^52^ we verified and calculated multiple possibilities for the Gompertz model for the growth rate α of deaths and infections, and with various values of the *k* parameter with the maximum expected number of infected and dead by country. Moreover, we took into account the different possibilities of the indicators (*p*, *d*, *q*) of the ARIMA model to obtain the values closest to the real data collected up to the date of the study (May 19, 2020). Based on the different results, we compared the predicted values with the actual values. To this aim, we worked out a quantitative examination of the fit using error measurement indices, commonly used to evaluate prediction models.^53^ We used Karl Pearson’s *R*^2^ regression index^54^ to justify its greater or lesser correlation.^55^ Additionally, we compared the model accuracy of the different regressions according to the RMSE^56^ and MAPE,^57^ which are forecast indicators that measure the size of the absolute error in percentage terms, giving us a relative measure of the error. The functions used for accuracy calculations are as follows:

(Eq. 5) $$\mathrm{RMSE}={\left\lfloor \frac{1}{t}\sum_{i=1}^{t}{\left(u_{p}-u_{o}\right)}^{2}\right\rfloor }^{1/2}$$

(Eq. 6) $$MAPE=\frac{100}{t}\sum_{i=1}^{t}\left|\frac{k_{r}-k_{f}}{k_{r}}\right|$$

where *t* denotes the number of observations, *u* is the residue of the estimates, the subscript *p* refers to the predicted residue whereas subscript *o* is the observed residue, *k_r_* is the actual number of infected or dead, and *k_f_* is the estimated number of infected or dead according to the analyzed prediction model.

## RESULTS

We worked out the maximum infected forecasts using the Gompertz and ARIMA models. The results suggest that the three countries are close to the end of the pandemic. It can be observed in the decrease in daily accumulated cases and the flattening of the curve in both deaths and infections. Table 1B shows the values obtained and the characteristic parameters of the pandemic. With the calculated data, it is observed that the prognosis obtained for Switzerland is for almost twice as many infected as for Austria and Israel, and a very high infection rate of 3,605 infected per million inhabitants, compared to the other two countries. Austria is the country with fewest infected, with an infection rate of half that of Switzerland and only 1,875 infected per million inhabitants. The forecast of the end of the pandemic has been estimated regarding the stabilization in the growth rates. The pandemic is predicted to end in June for Austria and one month later for Israel and Switzerland.

For this model to predict the growth in number of deaths from COVID-19, we used the Gompertz and ARIMA models, bearing in mind that the actual numbers of deaths are close to those predicted. Table 1C shows the data obtained, with the case fatality rate (CFR) and deaths per million inhabitants. It is observed that Israel has a CFR of 1.7% and 35 deaths per million, which is the lowest of the three countries studied. The highest CFR is again obtained by Switzerland, 3.8 times higher than that of Israel. Switzerland has a CFR of 6.5%, with deaths per million inhabitants 6.7 times higher than in Israel, at a value of 233 deaths per million inhabitants. Based on the results obtained, we would predict the end of the pandemic, estimated to the end of June for Austria and Israel, and in July for Switzerland.

Once the calculations of both ARIMA and Gompertz prediction models were performed, we obtained Table 1D and E for infected and dead, respectively. It can be seen that the ARIMA model has greater precision than the Gompertz model in both deceased and infected models, with only minor differences between their values. The main difference is that the Gompertz model is easier to use to estimate the completion of the pandemic than the ARIMA model.

**Figure 3:**
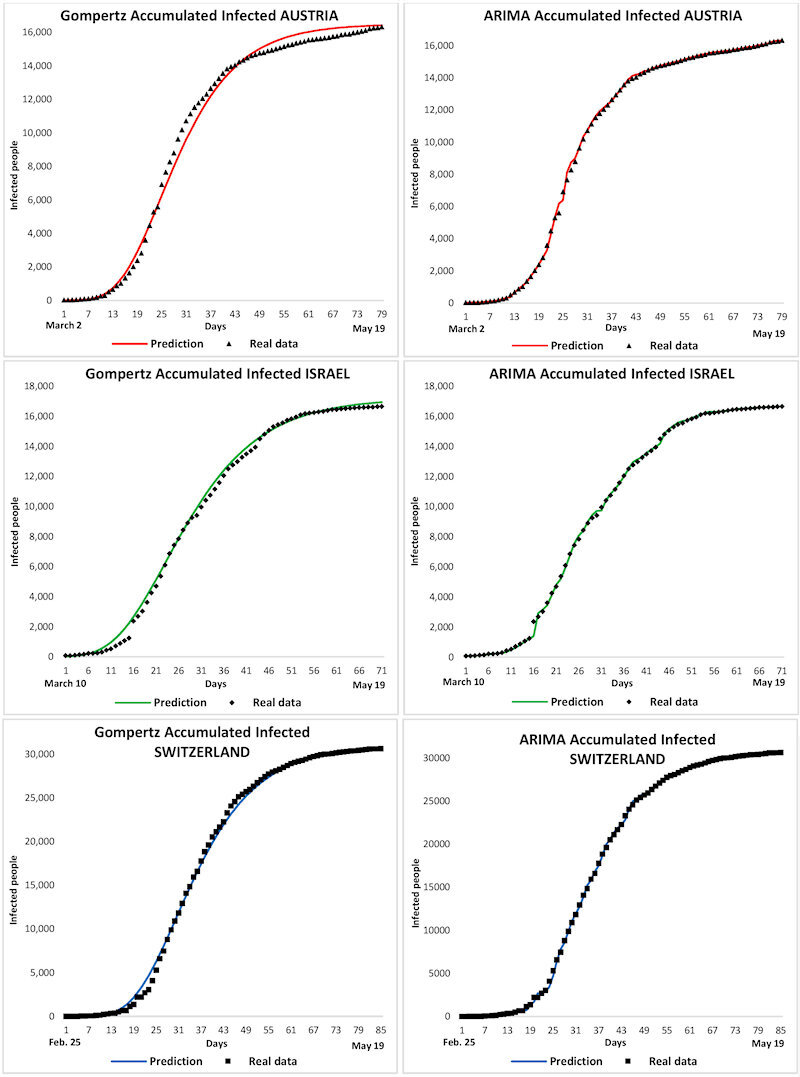
Gompertz and ARIMA Models for Accumulated Infected in Austria, Israel, and Switzerland.

**Figure 4:**
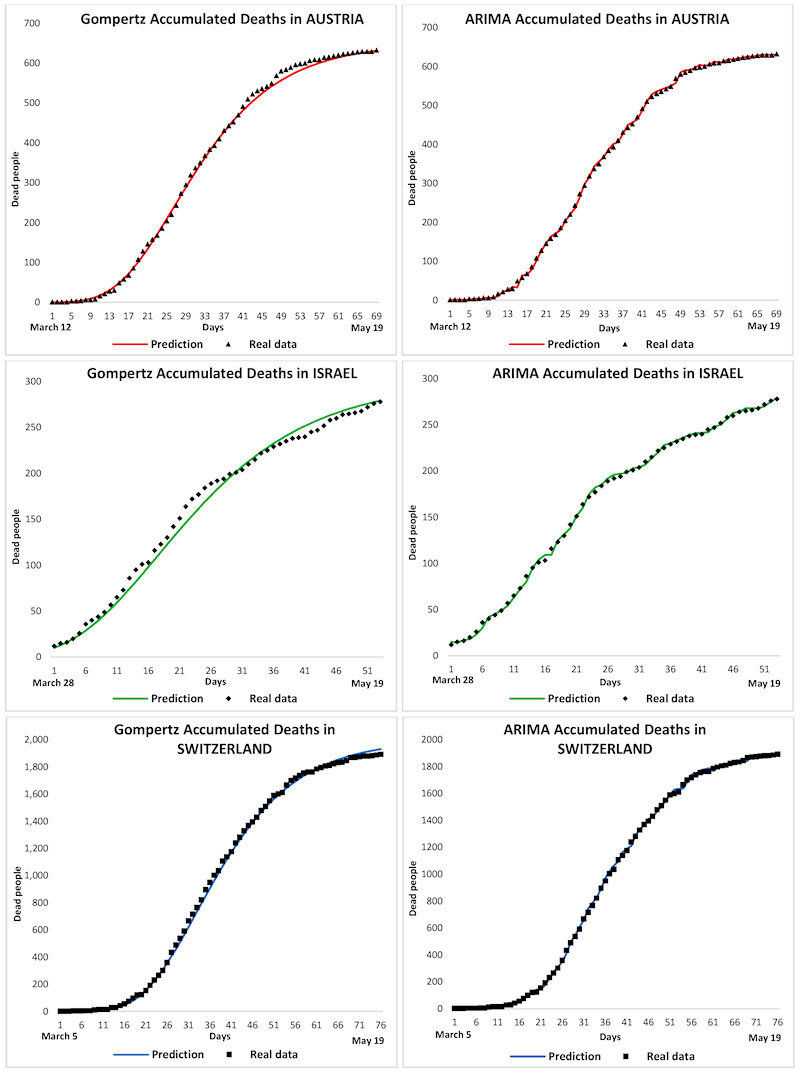
Gompertz and ARIMA Models for Deaths Accumulated in Austria, Israel, and Switzerland.

Figure 3 shows the accumulated number of infected individuals for both the Gompertz and the ARIMA models for the three studied countries. It is observed that the Gompertz model follows a sigmoid curve that predicts a less suitable contagion model than the ARIMA one. Indeed, the ARIMA model generates a statistical curve that better approaches the real data of contagion in the countries. Figure 4 shows the graphs of the accumulated deaths, where both models again match the actual data. It seems that the sigmoid curve of the Gompertz model is the one that shows a more realistic mortality curve. Nevertheless, the ARIMA statistical model achieves a better adjustment regarding real data.

## DISCUSSION

This work verifies the use of statistical growth and mortality models to analyze the growth and development of unexpected pandemics. Specifically, we show the performances when the Gompertz sigmoid curve growth model and the ARIMA statistical model are used. They seem to be suitable models to use with sudden and widespread outbreaks. Both simulation models present good results and excellent forecasts. In both models, the correlation index is higher than 0.999 in all cases. Also, we verified that the mortality forecasts have greater accuracy with the Gompertz model (MAPE<10%), and the ARIMA model is more accurate for modeling the number of infected individuals, in all three countries (MAPE<6%).

This study is valid for estimating deaths in the pandemic with regard to infected individuals who were detected with symptoms. However, the models do not take into account those individuals who were asymptomatic. The limitations shown by both models must also be taken into account in future predictions of the end of epidemics since growth indicators must be estimated for each investigation. Another significant limitation of the study is the external factors of containment of pandemics that are applied by each country, and a further limitation is that only confirmed cases can be compared and not asymptomatic cases, which could lead to new unexpected outbreaks due to patients that could not be detected. Such factors can cause growth patterns to decrease or increase significantly depending on whether the right measures are taken to contain cases and stop the spread of the virus. Meanwhile, it is worth pointing out the importance of obtaining reliable data from government health entities to make the correct mathematical predictions. With the values obtained from the statistical models, the end of the pandemic can be predicted, *if* they follow the measures adopted and there are no uncontrolled outbreaks. Control policies in countries must continue until the pandemic is eradicated in order to adequately protect the population.

## CONCLUSIONS

This study demonstrates the validity of the model based on the biological growth function of Gompertz and the statistical model of ARIMA to describe the pandemic growth of COVID-19 both in terms of the numbers of infections and deaths, and in terms of the dynamic progression to predict the point of the trend change. We verify that the models are adaptable to different countries with different socio-political circumstances, adjusting the growth and statistical coefficients for each case. The obtained results confirm that the theoretical curves are very close to the actual evolution of the pandemic. Thus, we achieved forecasts with high correlation coefficients concerning real cases, higher than 0.99 in both models, and in all the circumstances and all three countries studied. In the three countries Austria, Switzerland, and Israel, the restriction policies must be followed until the end of the pandemic, and control and follow-up of asymptomatic cases should be implemented in order to control possible outbreaks.

The objective of this work was to predict the date when a given country will reach the peak of the pandemic. To this end, both models took into account the maximum number of people affected by the pandemic, making it possible to describe the epidemiological stability rates based on rates obtained in different countries that have already reached the peak of the pandemic. Furthermore, both models studied in this paper can be extended to other affected countries to forecast the likely course of the disease. The authors hope that this project will be of help to health and political authorities during the difficult times of this global pandemic outbreak.

## Abbreviations

ARIMA: autoregressive integrated moving average
COVID-19: corona virus disease of 2019
CFR: case fatality rate
ECDC: European Center for Disease Prevention and Control
MAPE: mean absolute percentage error
RMSE: root mean squared error
SARS-CoV-2: severe acute respiratory syndrome coronavirus-2
WHO: World Health Organization.
